# Association of *GHRL* and *CYP19A1* Variants with Uterine Leiomyoma: An Exploratory Case–Control Study with In Silico Annotation

**DOI:** 10.3390/ijms27219625

**Published:** 2026-10-27

**Authors:** Eren Akbaba, Burak Sezgin, Ayşegül Demirtaş, Çilem Özdemir, Tuba Edgünlü

**Affiliations:** 1 Department of Gynaecology and Obstetrics, Faculty of Medicine, Muğla Sıtkı Koçman University, 48000 Mugla, Turkey; 2 Department of Medical Biology, Health Sciences Institution, Muğla Sıtkı Koçman University, 48000 Mugla, Turkey; 3 Department of Bioinformatics, Graduate School of Natural and Applied Sciences, Mugla Sıtkı Koçman University, 48000 Mugla, Turkey; 4 Department of Medical Biology, Faculty of Medicine, Muğla Sıtkı Koçman University, 48000 Mugla, Turkey

**Keywords:** uterine leiomyoma, *CYP19A1*, *GHRL*, gene polymorphism, rs2414096, rs34911341, rs696217, single-nucleotide polymorphism (SNP), aromatase

## Abstract

Uterine leiomyomas (ULMs) are common benign gynecological tumors associated with estrogen signaling and metabolic–endocrine pathways. This study investigated the associations of *CYP19A1* (cytochrome P450 family 19 subfamily A member 1; rs2414096) and *GHRL* (ghrelin and obestatin prepropeptide; rs34911341, rs696217) polymorphisms with leiomyoma risk and evaluated their potential functional effects in silico. This case–control study included 65 women with uterine leiomyoma and 72 healthy controls. Genotyping was performed using polymerase chain reaction–restriction fragment length polymorphism (PCR-RFLP). Associations were assessed using Firth’s penalized-likelihood logistic regression under additive, dominant, and recessive models, with adjustment for age, body mass index (BMI), and parity. Functional predictions employed CADD, PolyPhen-2, SIFT, SpliceAI, and I-Mutant v2.0. Cases and controls were comparable in age, BMI, and parity (all *p* > 0.05). *CYP19A1* rs2414096 was not associated with leiomyoma risk. Conversely, *GHRL* rs34911341 showed a significant association under the adjusted additive model (OR = 2.85, 95% CI: 1.59–5.45, *p* = 0.0003; FDR q = 0.001) and remained independently associated in the joint multivariable model (OR = 2.75, 95% CI: 1.52–5.30, *p* = 0.0006). *GHRL* rs696217 showed a nominal additive association (adjusted OR = 2.11, *p* = 0.037), which did not remain significant after false discovery rate (FDR) correction or in the joint model. Recessive associations for both *GHRL* variants were affected by complete separation. In silico analyses classified rs2414096 as benign, whereas both *GHRL* variants were computationally predicted to be potentially deleterious and to reduce protein stability. These findings suggest a candidate association between *GHRL* rs34911341 and leiomyoma susceptibility. Given the modest sample size, control-group Hardy–Weinberg equilibrium deviations, and absence of biochemical peptide measurements, larger multicenter studies are needed for validation.

## 1. Introduction

Uterine leiomyomas (fibroids) represent the most prevalent benign mesenchymal neoplasms of the female reproductive tract, clinically identified in up to 70–80% of women by the end of their reproductive years [1,2]. Characterized by an excessive proliferation of smooth muscle cells surrounded by an abundant extracellular matrix (ECM), these tumors constitute a major global public health concern [1,3]. Although non-malignant in nature, uterine leiomyomas are associated with substantial clinical morbidity, causing severe symptoms such as heavy or prolonged menstrual bleeding, pelvic pain and pressure, severe secondary anemia, and adverse reproductive outcomes, including recurrent pregnancy loss and infertility [1,3]. Consequently, leiomyomas remain the primary surgical indication for hysterectomy worldwide, imposing a significant financial and physical burden on healthcare systems [1,2].

The pathogenesis of uterine leiomyomas is multifaceted and complex, arising from an intricate interplay between somatic genetic mutations, epigenetic alterations, growth factor signaling, and localized endocrine dysfunction [2,4]. Among these mechanisms, steroidal hormone responsiveness—specifically centered on estrogen and progesterone—plays a central role in initiating and propagating leiomyoma expansion [4,5]. Estrogens promote cell proliferation and ECM accumulation predominantly through localized tissue bioavailability rather than systemic endocrine levels alone [2,5]. A critical mediator of local intracellular estrogen synthesis is the enzyme cytochrome P450 aromatase, encoded by the *CYP19A1* gene (located on chromosome 15q21.2). Unlike normal human myometrium, which exhibits minimal or completely undetectable aromatase expression, uterine leiomyomas aberrantly express functional aromatase at high levels [2,6]. This aberrant local expression leads to the in situ conversion of circulating C19 androgen precursors (such as androstenedione) into potent C18 estrogens (estrone and estradiol), establishing an autocrine and paracrine feedforward loop that accelerates tumor growth [2,6,7]. Genetic variation within the *CYP19A1* gene, including rs2414096, has therefore been investigated as a potential modifier of estrogen-related phenotypes and susceptibility to estrogen-dependent gynecological disorders [4,8].

**Figure 1:**
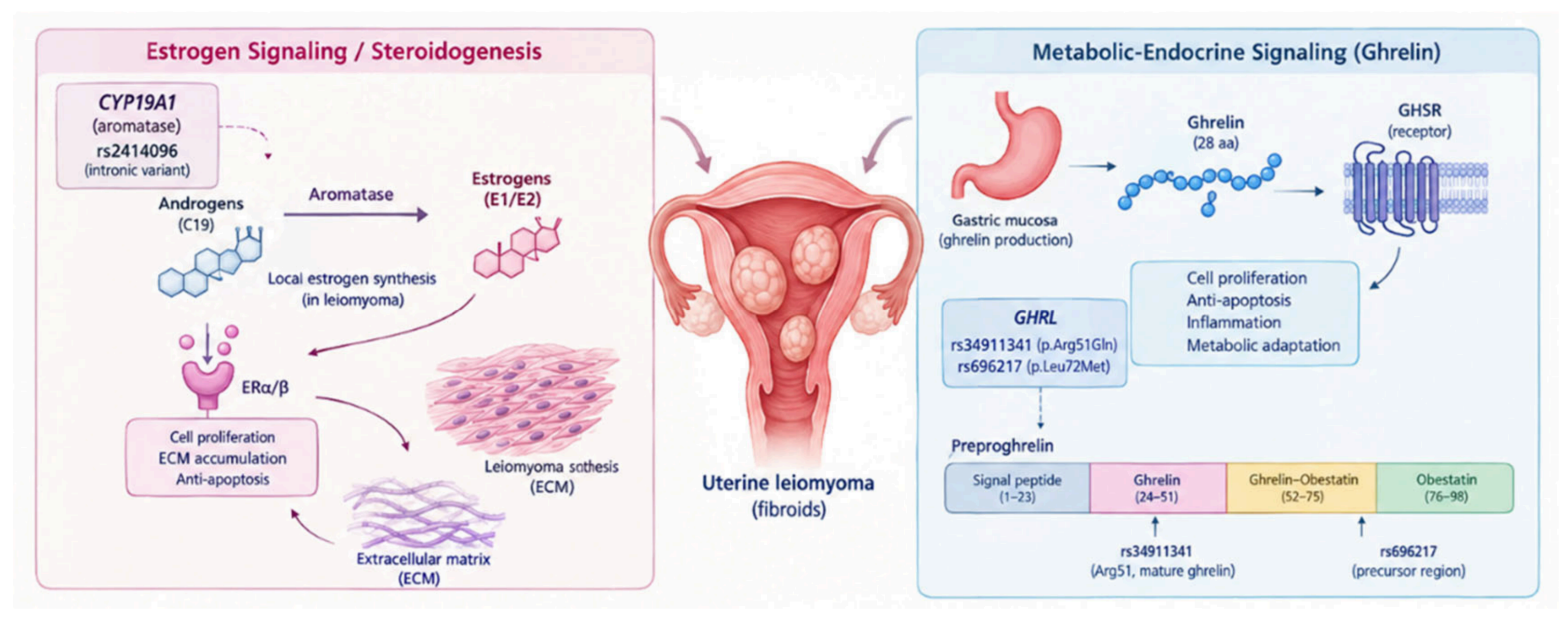
Schematic representation of the multifactorial pathogenesis of uterine leiomyoma, highlighting estrogen signaling and metabolic–endocrine pathways. Local estrogen synthesis mediated by aromatase and ghrelin-related signaling are presented as biologically relevant pathways potentially contributing to leiomyoma development. The selected *CYP19A1* (rs2414096) and *GHRL* (rs34911341 and rs696217) variants are indicated within their respective pathways. The figure provides the biological rationale for the candidate-variant approach and does not imply established causal effects of these variants on leiomyoma susceptibility.

Parallel to the classical steroidogenic hypothesis, emerging clinical and molecular evidence underscores a critical metabolic–endocrine crosstalk in fibroid pathophysiology. Metabolic dysregulation, body mass index (BMI), visceral adiposity, and systemic peptide signaling are increasingly recognized as potent modulators of the uterine microenvironment [3,9]. Within this framework, ghrelin (*GHRL*) is an orexigenic 28-amino-acid peptide hormone predominantly synthesized by gastric mucosal cells and is a key regulator of systemic energy homeostasis and growth hormone secretion. Operating through its functional receptor, the growth hormone secretagogue receptor (GHSR), ghrelin signaling extends well beyond appetite control to influence peripheral cell proliferation, anti-apoptotic cascades, chronic low-grade inflammation, and tissue-level metabolic adaptation [9,10]. Genetic variants within *GHRL*, including rs34911341 and rs696217, have been investigated in relation to metabolic phenotypes, circulating ghrelin-related traits, and cellular processes relevant to tissue growth [9,10]. However, their potential contribution to uterine leiomyoma susceptibility remains insufficiently characterized (Figure 1).

Uterine leiomyoma is highly prevalent and heritable, yet genetically heterogeneous. Genome-wide association studies (GWAS) in Japanese, European and multi-ancestry cohorts have identified multiple susceptibility loci, each with a modest per-allele effect (odds ratios (ORs) typically between 1.2 and 1.5) [11,12,13,14]. This is the expected architecture of a common multifactorial disease, in which susceptibility arises from many common variants of small effect, supplemented by rarer variants that may have larger effects but explain only a small fraction of cases [15]. We therefore do not assume that any single variant explains leiomyoma risk. Our aim was to test, in an exploratory candidate-variant design, whether selected variants in two biologically distinct pathways make a measurable contribution to susceptibility. The three variants were selected a priori based on their biological relevance and differences in population frequency according to Genome Aggregation Database (gnomAD) version 4.1.2 (European non-Finnish) [16]. *CYP19A1* rs2414096 (c.146-573C>T) is a common intronic variant (allele frequency ≈ 50.8%) in the gene encoding aromatase. Aromatase overexpression in leiomyoma is thought to be driven predominantly by alternative promoter usage, particularly promoters I.4 and II, and by tissue-specific transcriptional regulators such as CCAAT/enhancer-binding protein beta (C/EBP-β), rather than by germline coding variation [4]. Accordingly, rs2414096 was included as a common intronic variant with a potentially modest modulatory role within the steroidogenic pathway. *GHRL* rs34911341 (c.152G>A, p.Arg51Gln) is a lower-frequency coding missense variant (allele frequency ≈ 0.7%), whereas *GHRL* rs696217 (c.214C>A, p.Leu72Met) is a more common missense variant (allele frequency ≈ 8%) affecting the preproghrelin sequence. These variants were selected to capture genetic variation across estrogen-related steroidogenesis and ghrelin signaling while representing different allele-frequency contexts. Although common variants in complex traits may contribute modest effects that are difficult to detect in relatively small cohorts, coding missense variants may also have the potential to influence peptide processing, structure, or biological activity. Given the limited sample size, this single-centre study was designed as an exploratory, hypothesis-generating evaluation of these biologically related pathways rather than as a definitive estimate of population-level genetic effect sizes.

Given the established involvement of steroid hormone signaling and metabolic–endocrine regulation in uterine leiomyoma pathophysiology, we hypothesized that genetic variation in these biologically distinct pathways may contribute to interindividual susceptibility. Therefore, the primary aim of this case–control study was to investigate the genotype and allele distributions of *CYP19A1* rs2414096 and *GHRL* rs34911341 and rs696217 in patients with uterine leiomyoma and healthy controls and to evaluate their potential associations with disease susceptibility under different genetic inheritance models. The study was designed as an exploratory, hypothesis-generating genetic association analysis rather than a clinical risk-prediction study. To our knowledge, this is the first study to jointly investigate *CYP19A1* and *GHRL* polymorphisms, representing steroidogenic and metabolic–endocrine pathways, respectively, in the context of uterine leiomyoma, complemented by in silico functional analyses.

## 2. Results

### 2.1. Study Population

The baseline demographic and clinical characteristics of the case and control groups are summarized in Table 1. The median age was 44.76 years (IQR: 39.17–48.31; range: 27.04–63.15 years) in the case group (*n* = 65) and 43.21 years (IQR: 40.55–47.36; range: 31.27–56.39 years) in the control group (*n* = 72), with no statistically significant difference between the groups (*p* = 0.574). Similarly, BMI did not differ significantly between cases and controls [median 26.84 kg/m^2^ (IQR: 25.37–28.93) vs. 25.75 kg/m^2^ (IQR: 22.63–29.06), *p* = 0.096]. Parity distributions were likewise comparable between groups when evaluated as a continuous metric [median 2 (IQR: 1–2) in both groups, *p* = 0.189] or as categorical frequencies (*p* = 0.184). Baseline differences were not statistically significant; because this does not exclude confounding, all genetic associations were additionally adjusted for age, BMI and parity.

### 2.2. Genotype and Allele Distributions

The genotype and allele distributions of the *CYP19A1* rs2414096, *GHRL* rs34911341, and *GHRL* rs696217 polymorphisms were compared between the patient and control groups (Table 2).

For *CYP19A1* rs2414096, no significant difference was observed in genotype distribution between patients and controls (*p* = 0.565). Similarly, allele frequencies did not differ significantly between the two groups (*p* = 0.369).

In contrast, the genotype distribution of *GHRL* rs34911341 differed significantly between patients and controls (*p* < 0.001). The TT genotype was observed in 32.3% of patients but was absent in controls, whereas the TC genotype was more frequent among controls than patients (72.2% vs. 46.2%). The allele distribution also differed significantly between the groups (*p* = 0.002), with the T allele being more frequent in patients than in controls (55.4% vs. 36.1%).

The genotype distribution of *GHRL* rs696217 also differed significantly between the two groups (*p* < 0.001). The GG genotype was observed in 21.5% of patients but was absent in controls, whereas the TG genotype was more frequent among controls than patients (87.5% vs. 61.5%). However, allele frequencies did not differ significantly between patients and controls (*p* = 0.183), with the G allele showing an OR of 1.41 (95% CI: 0.88–2.27).

Overall, *GHRL* rs34911341 and rs696217 showed significant differences in genotype distributions between patients and controls, whereas *CYP19A1* rs2414096 showed no significant difference in either genotype or allele distribution. Notably, the significant genotype-level difference observed for *GHRL* rs696217 was not reflected at the allele level.

After correction for the six primary tests (Holm), the genotype-level associations for *GHRL* rs34911341 (adjusted *p* = 5.9 × 10^−6^) and rs696217 (adjusted *p* = 3.7 × 10^−4^) and the allele-level association for rs34911341 (adjusted *p* = 0.006) remained significant, whereas the rs696217 allele test (adjusted *p* = 0.55) and all *CYP19A1* tests (adjusted *p* ≥ 0.74) did not. Among the nine model-based tests, the additive and recessive models for rs34911341 and the recessive model for rs696217 remained significant at FDR q < 0.05, whereas the additive model for rs696217 did not (q = 0.10). These multiplicity-adjusted findings should nevertheless be interpreted cautiously, particularly for the *GHRL* variants, given the sparse genotype cells and marked deviations from Hardy–Weinberg equilibrium observed in the control group.

### 2.3. Hardy–Weinberg Equilibrium and Statistical Precision

Genotype distributions of *CYP19A1* rs2414096 complied with HWE in both controls (exact *p* = 1.00) and cases (*p* = 0.55). In contrast, controls exhibited marked deviation from HWE for *GHRL* rs34911341 (exact *p* = 3.0 × 10^−7^) and rs696217 (*p* = 2.4 × 10^−12^), characterized by an excess of heterozygotes and absence of one homozygote class (TT and GG, respectively), whereas cases remained consistent with HWE (*p* = 0.62 and *p* = 0.084). To evaluate assay reproducibility, duplicate PCR-RFLP genotyping was performed on a random 10% subset of samples by an independent blinded operator, showing 100% concordance with the primary assay. The ORs for the TT (rs34911341) and GG (rs696217) genotypes, which were absent in controls, were estimated using the Haldane–Anscombe continuity correction (OR = 60.8, 95% CI: 3.4–1086.7, and OR = 24.0, 95% CI: 1.26–456.5, respectively) and are reported as descriptive estimates only. The sensitivity analysis indicated that, with 65 cases and 72 controls, the study achieved 80% statistical power only for detecting large effect sizes: allele-level OR ≥ 2.0 (or ≤0.47), and dominant-model OR ≥ 2.9 for *CYP19A1*, ≥3.6 for rs34911341, and ≥ 9.6 for rs696217 (carrier frequencies in controls: 58%, 72%, and 88%, respectively). Furthermore, the 95% confidence interval for the *CYP19A1* allele OR (0.78, 95% CI: 0.47–1.30) remains compatible with both a 50% reduction and a 30% increase in odds.

### 2.4. Model-Based Association and Joint Multivariable Analysis

In Firth logistic regression, the rs34911341 T allele was associated with leiomyoma under the additive model (OR = 2.83, 95% CI 1.59–5.35, *p* = 0.0003). The association was unchanged after adjustment for age, BMI and parity (OR = 2.85, 95% CI 1.59–5.45, *p* = 0.0003), and it was not significant under the dominant model (adjusted OR = 1.42, 95% CI 0.66–3.16) (Table 3).

Under the recessive model, the TT genotype was observed only in cases, so the estimate was driven by a zero cell and is imprecise (adjusted OR = 69.4, 95% CI 9.0–8829); it should be read as indicating the direction of the association rather than its size. For rs696217, the G allele showed a weaker additive association (adjusted OR = 2.11, 95% CI 1.04–4.55, *p* = 0.037; FDR q = 0.084) with the same zero-cell limitation under the recessive model and no association under the dominant model (adjusted OR = 0.79, 95% CI 0.30–2.06). *CYP19A1* rs2414096 showed no association under any model (additive adjusted OR = 0.79, 95% CI 0.48–1.29).

In a joint model including all three variants, age, BMI and parity, only rs34911341 remained associated with leiomyoma (OR = 2.75 per T allele, 95% CI 1.52–5.30, *p* = 0.0006), whereas rs696217 (OR = 1.90, 95% CI 0.82–4.57, *p* = 0.13) and *CYP19A1* (OR = 0.94, 95% CI 0.52–1.66) did not contribute independent information. Age, BMI and parity were not significantly associated with leiomyoma in this model (Table 4).

### 2.5. In Silico Functional and Regulatory Analysis

Multi-tool in silico analysis revealed distinct predicted functional profiles among the three investigated polymorphisms (*CYP19A1* rs2414096, *GHRL* rs696217, and *GHRL* rs34911341). None of the variants were predicted to have a significant effect on pre-mRNA splicing by SpliceAI and Pangolin (Δ ≤ 0.02).

The intronic *CYP19A1* rs2414096 variant showed limited predicted functional impact, with a low CADD score (2.6) and weak evolutionary conservation (PhyloP = 0.846). Chromatin-state annotations also indicated predominantly quiescent or Polycomb-repressed chromatin states.

In contrast, *GHRL* rs696217 (p.Leu72Met) showed high conservation (PhyloP = 5.81), elevated CADD (23.2), and a near-maximal PolyPhen-2 score (0.999), although SIFT predicted a tolerated effect (0.10). I-Mutant v2.0 predicted reduced protein stability (reliability index [RI] = 8). Similarly, *GHRL* rs34911341 (p.Arg51Gln) showed high conservation (PhyloP = 5.98), high CADD (25.0), high PolyPhen-2 score (0.998), and a borderline deleterious SIFT score (0.05), together with reduced predicted protein stability (RI = 5). Both variants were located in weak transcriptional chromatin states without evidence of altered splicing. Overall, the computational findings suggest greater functional relevance for the two *GHRL* coding variants, particularly rs34911341, whereas rs2414096 showed minimal predicted functional impact. The results of the in silico functional and regulatory analyses are summarized in Table 5.

## 3. Discussion

The present study investigated the genotype and allele distributions of *CYP19A1* rs2414096, *GHRL* rs34911341 (Arg51Gln), and *GHRL* rs696217 (Leu72Met) in women with uterine leiomyoma and disease-free controls, complemented by an in silico functional characterization of the three variants. Table 6 summarises the principal findings, their biological interpretation and the main limitations, which are discussed in detail below.

We found no significant difference in the genotype or allele distribution of *CYP19A1* rs2414096 between cases and controls. Aromatase is a key driver of local estrogen biosynthesis in leiomyoma tissue [2], yet a case–control study of Brazilian women also found no difference in *CYP19* or *CYP17A1* genotype and allele frequencies between patients and controls, concluding that steroidogenic gene polymorphisms are unlikely to be major contributors to susceptibility [17]. Our result is consistent with this. Associations of rs2414096 with genotype distribution and hyperandrogenism markers have been described mainly in polycystic ovary syndrome (PCOS) [18,19]; this discrepancy may reflect disease-specific pathophysiology, as PCOS is driven by systemic hyperandrogenism, whereas leiomyoma growth depends more on local, tissue-level estrogen production. The in silico profile of rs2414096 (low CADD score, weak conservation, no predicted splicing effect, and quiescent or Polycomb-repressed chromatin) is consistent with a benign, non-disruptive polymorphism.

In contrast, both *GHRL* variants showed associations in model-based analyses. In adjusted Firth logistic regression, the rs34911341 T allele was significantly associated with leiomyoma risk under the additive model (adjusted OR = 2.85, *p* = 0.0003; FDR q = 0.001) and remained the sole independent genetic predictor in a joint model including all three variants, age, BMI and parity (OR = 2.75, *p* = 0.0006). rs696217 showed only a nominal additive association (adjusted OR = 2.11, *p* = 0.037) that did not survive FDR correction (q = 0.084) and had no independent effect in the joint model (OR = 1.90, *p* = 0.133). The two *GHRL* loci were weakly correlated (Spearman r = 0.12, *p* = 0.170), excluding collinearity. These findings are biologically plausible: the ghrelin/GHS-R axis operates locally in human myometrium and leiomyoma through autocrine/paracrine signalling modulated by estradiol and inflammatory cascades [22], and elevated active ghrelin and active-to-total ghrelin ratios in women with leiomyoma point to altered post-translational processing rather than simple overproduction [20,21]. rs34911341 (Arg51Gln) lies at the C-terminal cleavage boundary of mature ghrelin (residues 24–51), providing a plausible, though experimentally untested, basis for altered processing. Although Arg51Gln was first identified in obese individuals [33] and linked to lower plasma ghrelin and metabolic traits [34], our models were adjusted for continuous BMI in a cohort that included women with obesity (BMI ≥ 30 kg/m^2^), indicating that the association is independent of body mass. rs696217 (Leu72Met) lies in the preproghrelin region between ghrelin and obestatin (residues 76–98), where any effect more likely involves precursor stability or cleavage than mature peptide structure. Its metabolic associations are inconsistent across populations [23,24,25] (Table 7), which parallels the discrepancy we observed between genotype-level (significant) and allele-level (non-significant) results.

In silico predictions suggest that both *GHRL* missense variants may be functionally relevant, but they are computational predictions, not experimental measurements. rs34911341 was predicted to be deleterious by CADD and PolyPhen-2 (SIFT at the 0.05 threshold) and to reduce protein stability (I-Mutant v2.0), and because Arg51 is the C-terminal residue of mature ghrelin, the substitution could in principle alter peptide processing [33,40]. For rs696217, the stability prediction refers to the preproprotein, not mature ghrelin, and any effect would likely involve proghrelin stability, endoproteolytic processing or obestatin maturation. Because circulating ghrelin was not measured, the genotype–ghrelin link remains inferential and hypothesis-generating.

With only three candidate variants and no ghrelin measurements, our data do not permit conclusions on the relative importance of metabolic versus steroidogenic pathways in leiomyoma aetiology. The findings pertain strictly to the variants studied (an independent association for *GHRL* rs34911341, none for *CYP19A1* rs2414096) and should be regarded as variant-specific, exploratory observations rather than evidence of differential pathway dominance.

To our knowledge, no previous study has examined these three variants in uterine leiomyoma in Turkish or neighbouring populations, which limits direct regional comparison. Available Turkish data concern other candidate loci and indicate locus-specific rather than uniform effects [26,27,28]; together with our null *CYP19A1* result, they suggest that estrogen-pathway variants do not consistently influence leiomyoma susceptibility in this population. Regional *GHRL* data derive almost exclusively from metabolic phenotypes [29,30], and reported 51Gln and 72Met frequencies vary markedly between populations [30,32] (Table 7). Our control frequencies exceed even these estimates, which we address as a limitation; replication in independent Turkish cohorts is needed.

The genotype classes absent in controls, *GHRL* rs34911341 TT (Gln51Gln) and rs696217 GG (Met72Met), deserve separate comment. Gln51 is rare in most reference populations (≈0.02 in German cohorts [31]; not detected in Han Chinese [32,38]), so Gln51Gln homozygotes are seldom analysed, and evidence for Gln51 comes only from metabolic phenotypes [30,34]. A homozygous substitution could in principle affect ghrelin processing on both alleles, but this is speculative and untested in uterine tissue. Conversely, Leu72Leu is the most common genotype in reference populations (Met72 ≈ 0.09 in German [31] and 0.12–0.17 in Han Chinese cohorts [32]); its complete absence from our controls, together with the excess of heterozygotes, more plausibly reflects sampling or genotyping artefacts than biology. *GHRL* variants have been studied mainly outside leiomyoma, with no association with PCOS [38] and a weak association of rs171407 with breast cancer risk [39]. No published study has linked these genotype classes to leiomyoma, so our TT and GG observations remain hypothesis-generating until confirmed in larger, independently genotyped cohorts.

The main limitations are summarised in Table 7. First, the modest sample size (65 cases, 72 controls) limits power, particularly for rarer genotypes such as *CYP19A1* AA, and for detecting the modest effects (OR ≈ 1.2–1.5) reported in leiomyoma GWAS [11,12]; the null *CYP19A1* finding is therefore inconclusive rather than evidence of no association. Second, the absence of the TT and GG genotypes in controls produces sparse cells and unstable, potentially inflated estimates [41], reflected in very wide confidence intervals. Orthogonal sequencing validation (e.g., Sanger) was not possible; despite concordant duplicate PCR-RFLP results, subtle genotyping artefacts or incomplete digestion cannot be excluded. The marked deviation from Hardy–Weinberg equilibrium in controls further limits interpretation of the genotype-level associations. Third, circulating ghrelin was not measured, so functional inferences rest on in silico predictions. In silico predictions suggest that the two *GHRL* missense variants may be functionally relevant, but they are computational predictions, not experimental measurements of stability or function. rs34911341 (p.Arg51Gln) was predicted to be deleterious by CADD and PolyPhen-2 (SIFT score at the 0.05 threshold) and to reduce protein stability by I-Mutant v2.0; Arg51 is the C-terminal residue of the mature ghrelin peptide (residues 24–51), so the substitution could in principle alter peptide processing [42,43]. rs696217 (p.Leu72Met) lies outside mature ghrelin, in the region of preproghrelin between ghrelin and obestatin (residues 76–98); the I-Mutant stability prediction therefore refers to the precursor (preproprotein), not to the mature peptide, and any potential effect likely involves proghrelin stability, endoproteolytic processing, or obestatin maturation. Because circulating ghrelin was not measured, the link between genotype and ghrelin biology remains inferential, and these predictions should be regarded as hypothesis-generating. The minor-allele frequencies in our controls are markedly higher than in reference databases and in a Turkish–Cypriot control group [29]; differences of this size are unlikely to reflect population structure and, together with the heterozygote excess, raise the possibility of systematic genotype misclassification. We therefore interpret the *GHRL* results as provisional. Finally, detailed fibroid characteristics—such as number, size, FIGO anatomical location, and clinical symptoms—were not systematically recorded for the case group, which limits our ability to evaluate genotype–phenotype correlations stratified by tumor burden or presentation.

Future multi-marker analyses combining genetic markers, circulating ghrelin and clinical variables will require a different framework from the present comparison of three pre-specified variants, namely simultaneous multivariable modelling, variable selection with control of false discoveries, and independent validation or cross-validation following prediction-model reporting guidelines [44].

Penalised regression (LASSO, elastic net) or Bayesian spike-and-slab selection [45,46] would be suitable, with Firth-type penalisation for rare genotype classes. We did not apply such methods because only three variants were tested and the sample is too small to support variable selection. Accordingly, single-SNP genotyping based on the present findings is not suitable for clinical prediction or risk stratification.

## 4. Materials and Methods

### 4.1. Study Population and Sample Collection

This study was conducted among women over 18 years of age who presented to the Department of Obstetrics and Gynecology Outpatient Clinic at Muğla Training and Research Hospital. Based on clinical and imaging findings, 65 women from reproductive age through the menopausal transition diagnosed with uterine leiomyoma (ULM) were included in the case group. For comparison, 72 healthy women without ULM spanning a similar age range were recruited from the same clinic and geographical region during the same observation period to serve as a control group, yielding a total study population of 137 participants. Controls were selected to mirror the age distribution of the case group to achieve a comparable demographic profile, and age was subsequently included as a covariate in all multivariable models to adjust for potential residual confounding. Controls were defined by ultrasonography only. All examinations were performed by a single experienced gynaecologist using a GE Voluson 730 Expert ultrasound system (GE Healthcare, Chicago, Illinois, USA) with transabdominal (4–9 MHz) and transvaginal (3.7–9.3 MHz) probes. Fibroids were defined as well-circumscribed myometrial masses ≥ 10 mm in maximum diameter in accordance with the Morphological Uterus Sonographic Assessment (MUSA) terminology [47]. Controls were confirmed to have a normal uterus with no myometrial masses using the identical protocol. No formal individual matching was performed; controls were frequency-matched to cases on age to achieve a comparable demographic distribution.

Demographic and clinical parameters, including age, BMI, and parity, were systematically recorded for all subjects. Pregnant women, individuals with a history of malignant disease, diabetes mellitus, thyroid disorders, prior oral contraceptive use, or previous myomectomy were excluded from the study. These exclusion criteria were applied because pregnancy, diabetes mellitus, thyroid dysfunction, exogenous hormone exposure, and prior myomectomy are all established confounders of circulating and local estrogen and ghrelin levels and could therefore independently influence genotype–phenotype associations unrelated to the polymorphisms under investigation. Obesity was not an exclusion criterion; women of all BMI categories were enrolled, and BMI was incorporated as a continuous covariate in all multivariable association models to adjust for adiposity.

Deviation from Hardy–Weinberg equilibrium (HWE) was assessed separately in controls and cases using the exact test [42]; deviation in controls was interpreted as a possible indicator of genotyping error [48,49]. Because post hoc power calculations are uninformative for completed studies [50,51], we report 95% confidence intervals as measures of precision and a sensitivity analysis giving the smallest odds ratio detectable with 80% power (two-sided α = 0.05) for 65 cases and 72 controls at the observed control genotype and allele frequencies. For genotype categories with zero counts in one group, the odds ratio was not estimable by the usual formula; we applied the Haldane–Anscombe correction (0.5 added to every cell) and, in regression models, Firth’s penalised likelihood [43,52]. Such estimates are reported as descriptive only. All participants were unrelated; no first- or second-degree relatives were included.

For genetic analyses, a 2 mL venous blood sample was collected from each participant during routine clinical sampling and transferred into tubes containing 2% ethylenediaminetetraacetic acid (EDTA). The study protocol was approved by the Clinical Research Ethics Committee of Muğla Sıtkı Koçman University (Date: 6 January 2021, Decision No: 1/IV).

### 4.2. DNA Extraction and Genotyping

Venous blood samples (2 mL) were collected in vacutainer tubes containing sodium/potassium EDTA. DNA was extracted with a Hibrigen blood DNA isolation kit (MG-KDNA-02–250; Hibrigen Biotechnology R&D Industry and Trade Inc., Gebze, Kocaeli, Turkey). The *CYP19A1* (rs2414096) and *GHRL* (rs34911341 and rs696217) polymorphisms were determined by the polymerase chain reaction (PCR) restriction fragment length polymorphism (RFLP) method. PCR was performed in a 25-µL volume with 100 ng of DNA and 2X Taq Master Mix (MG-TAQMX-01–80; Hibrigen Biotechnology R&D Industry and Trade Inc., Gebze, Kocaeli, Turkey) using an automated thermal cycler (SimpliAmp Thermal Cycler, Thermo Fisher Scientific, Waltham, MA, USA). The primer sequences, PCR annealing temperature, and RFLP conditions are listed in Table 8. The alleles and single-nucleotide polymorphisms evaluated in this study were verified against dbSNP and gnomAD databases, and tabulated alleles refer to the positive reference genomic strand.

All 137 samples were successfully genotyped for the three variants with a 100% call rate. Agarose gels were scored independently by two readers blinded to case–control status. To ensure technical reliability and rule out incomplete enzymatic cleavage, every gel run incorporated an undigested PCR product as a negative cleavage control alongside a known homozygous reference sample where available. Furthermore, re-analysis of a random 10% subset (*n* = 14) by an independent blinded operator yielded 100% concordance.

### 4.3. In Silico Functional and Regulatory Annotation

In silico functional characterization of the three candidate polymorphisms (*CYP19A1* rs2414096, *GHRL* rs696217, and *GHRL* rs34911341) was performed using a suite of publicly available web-based bioinformatics tools.

Splicing effects were predicted with SpliceAI [v1.3.1] [53] and Pangolin [v1.0.2] [54] using the SpliceAI Lookup web server (https://spliceailookup.broadinstitute.org; Gencode v50 accessed on 20 August 2026); a maximum delta score ≤ 0.02 was classified as no predicted effect, which is far below the cut-offs recommended by the SpliceAI authors (0.2, 0.5 and 0.8). The “Other predictors” panel of SpliceAI Lookup was used to obtain precomputed CADD [v1.7] [35], PhyloP [v1.9.9] [36], PolyPhen-2 [v2.2.3] [37] and SIFT [v1.2] [55] scores. Variants were interpreted using established thresholds of CADD ≥ 20, PhyloP > 2, PolyPhen-2 ≥ 0.95, and SIFT < 0.05.

The effect of rs34911341 and rs696217 missense substitutions on stability was assessed with I-Mutant2.0 [v4.0] [56] using the full-length preproghrelin sequence (UniProt Q9UBU3) at pH 7.0 and 25 °C, reporting the direction of change and the Reliability Index.

Regulatory potential of each variant was assessed using RegulomeDB version 2 (v2; https://regulomedb.org/) (accessed on 10 August 2026), which integrates ENCODE ChIP-seq, DNase I hypersensitivity, and chromatin-state segmentation data to assign a categorical regulatory classification [57], queried for Ishikawa endometrial cells, HeLa-S3 cells, and primary uterine tissue.

### 4.4. Statistical Analysis

All statistical analyses were performed using R statistical software (version 4.3.2; R Foundation for Statistical Computing, Vienna, Austria). Continuous variables (age and BMI) and discrete count variables (parity) were summarized as median (interquartile range, Q1–Q3) and compared between the case and control groups using the non-parametric Mann–Whitney U test. Parity distributions were additionally expressed as frequencies and percentages (*n*, %) and compared using the Fisher–Freeman–Halton exact test with Monte Carlo estimation. Normality was evaluated using the Shapiro–Wilk test.

Differences in genotype and allele distributions between groups were evaluated using Pearson’s Chi-square (chi^2^) test or Fisher’s exact test when expected cell counts were less than 5. For each variant, the reference allele was defined as the major allele in controls (*CYP19A1* G, rs34911341 C, rs696217 T), and the alternative (risk) allele as the minor allele. Unadjusted ORs with 95% Confidence Intervals (CIs) compared each non-reference genotype with the reference homozygote, and allelic ORs compared the alternative allele with the reference allele. For descriptive contingency table categories containing zero cell counts, the Haldane–Anscombe continuity correction was applied [58]. Genetic associations were formally assessed by logistic regression under additive (coded 0, 1, or 2 for the number of alternative alleles), dominant (heterozygote + alternative homozygote vs. reference homozygote), and recessive (alternative homozygote vs. others) inheritance models [59,60]. Because the true mode of inheritance is unknown a priori, all three models were evaluated for each variant; the additive model served as the primary model, while dominant and recessive models were secondary. Each model was fitted both unadjusted and after adjustment for clinically relevant covariates (age, BMI, and parity). Because two genotype classes (rs34911341 TT and rs696217 GG) were completely absent in controls—causing complete separation and non-finite maximum-likelihood estimates—Firth’s penalised-likelihood logistic regression with profile-likelihood confidence intervals and likelihood-ratio *p*-values was implemented for all model-based analyses [43,52]. Furthermore, a joint multivariable model incorporating all three variants (additive coding) along with age, BMI, and parity was fitted to assess whether the genetic variants carry mutually independent predictive information.

Because the sample size was fixed by clinic availability, we did not perform an a priori power calculation. Instead, a sensitivity analysis (two-sided α = 0.05, 65 cases, 72 controls, normal approximation for two independent proportions, observed control genotype frequencies) showed that the study had 80% power only for large effects: allelic OR ≥ 2.0, and dominant-model OR ≥ 2.9 for *CYP19A1* rs2414096, ≥3.6 for *GHRL* rs34911341, and ≥9.6 for *GHRL* rs696217 (carrier frequencies in controls 58%, 72% and 88%, respectively). Effects of the size reported in leiomyoma GWAS (OR ≈ 1.2–1.5) [11,12] could therefore not be reliably detected, and non-significant results, particularly for *CYP19A1*, should be read as inconclusive.

All analyses were treated as exploratory. The primary statistical family comprised the six unadjusted genotype- and allele-level tests, and *p*-values were adjusted using the Holm procedure [61]. The nine model-based tests (three variants × additive, dominant, and recessive models; Firth likelihood-ratio *p*-values) constituted a secondary family controlled by the Benjamini–Hochberg FDR procedure (q-values) [62]. A two-tailed *p*-value of < 0.05 was considered statistically significant.

## 5. Conclusions

In this exploratory case–control study, *GHRL* rs34911341 (and, less robustly, rs696217) showed genotype-level associations with uterine leiomyoma, whereas *CYP19A1* rs2414096 did not; the study was too small to exclude modest effects of any of the variants. In silico analyses predict a possible functional relevance of the *GHRL* missense variants, but this remains unverified. The data do not allow conclusions on the relative importance of the steroidogenic and metabolic–endocrine pathways or on interactions between them. Given the sample size, the deviation from Hardy–Weinberg equilibrium in controls and the lack of independent validation, the findings should be confirmed in larger independent cohorts with orthogonal genotyping and, ideally, circulating ghrelin measurements, before any clinical or mechanistic conclusions are drawn.

## Figures and Tables

**Table 1:** Baseline Demographic and Clinical Characteristics of the Study Participants.

Characteristic	Case (*n* = 65)	Control (*n* = 72)	*p*
Age, years, median (Q1–Q3)	44.76 (39.17–48.31)	43.21 (40.55–47.36)	0.574 ^a^
Age, range	27.04–63.15	31.27–56.39	
BMI, kg/m^2^, median (Q1–Q3)	26.84 (25.37–28.93)	25.75 (22.63–29.06)	0.096 ^a^
Parity, median (Q1–Q3)	2 (1–2)	2 (1–2)	0.189 ^a^
Parity, *n* (%)			0.184 ^b^
0	4 (6.2)	4 (5.6)	
1	22 (33.8)	29 (40.3)	
2	25 (38.5)	33 (45.8)	
≥3	14 (21.5)	6 (8.3)	

^a^ Mann–Whitney U test. ^b^ Fisher–Freeman–Halton exact test (Monte Carlo, 20,000 permutations). Parity ≥ 3 combines parity 3 (12 vs. 6) and 4 (2 vs. 0).

**Table 2:** The genotype and allele frequencies of the *CYP19A1* (rs2414096) and *GHRL* (rs34911341 and rs696217) gene polymorphisms in patients and controls.

Genotype/Allele	Control *n* (%)	Case *n* (%)	*p*	OR (95% CI)
*CYP19A1* rs2414096				
GG	30 (41.7)	33 (50.8)	0.565 (genotype)	Reference
GA	33 (45.8)	25 (38.5)		0.69 (0.34–1.41)
AA	9 (12.5)	7 (10.8)		0.71 (0.23–2.13)
Allele G	93 (64.6)	91 (70.0)	0.369 (allele)	Reference
Allele A	51 (35.4)	39 (30.0)		0.78 (0.47–1.30)
*GHRL* rs34911341				
CC	20 (27.8)	14 (21.5)	**<0.001** (genotype)	Reference
TC	52 (72.2)	30 (46.2)		0.82 (0.36–1.87)
TT	0 (0.0)	21 (32.3)		**60.8 †** (3.4–1086.7)
Allele C	92 (63.9)	58 (44.6)	**0.002** (allele)	Reference
Allele T	52 (36.1)	72 (55.4)		**2.20** (1.35–3.57)
*GHRL* rs696217				
TT	9 (12.5)	11 (16.9)	**<0.001** (genotype)	Reference
TG	63 (87.5)	40 (61.5)		0.52 (0.20–1.37)
GG	0 (0.0)	14 (21.5)		**24.0 †** (1.26–456.5)
Allele T	81 (56.3)	62 (47.7)	0.183 (allele)	Reference
Allele G	63 (43.8)	68 (52.3)		1.41 (0.88–2.27)

OR, odds ratio (odds of leiomyoma relative to the reference category); CI, confidence interval. † Standard OR could not be calculated because the genotype was absent in controls; the value shown was obtained using the Haldane–Anscombe correction (+0.5 added to each cell) and should be interpreted as descriptive only due to the extremely wide confidence intervals and estimate instability. Genotype *p*-values were derived from Pearson’s chi^2^ test; allele *p*-values were derived from Fisher’s exact test. Unadjusted *p*-values < 0.05 are shown in bold.

**Table 3:** Firth Penalised Logistic Regression Analysis of Genetic Association Models.

Variant (Alt. Allele)	Model	Crude OR (95% CI); p	Adjusted ^c^ OR (95% CI); p	FDR q (adj.)
*CYP19A1* rs2414096 (A)	Additive	0.79 (0.48–1.30); 0.356	0.79 (0.48–1.29); 0.344	0.48
	Dominant	0.70 (0.35–1.36); 0.289	0.70 (0.36–1.38); 0.303	0.48
	Recessive	0.86 (0.30–2.38); 0.767	0.81 (0.28–2.28); 0.695	0.69
*GHRL* rs34911341 (T)	Additive	2.83 (1.59–5.35); 0.0003	2.85 (1.59–5.45); 0.0003	0.001
	Dominant	1.39 (0.64–3.05); 0.406	1.42 (0.66–3.16); 0.373	0.48
	Recessive ^d^	70.1 (9.3–8903); 1.4 × 10^−8^	69.4 (9.0–8829); 2.7 × 10^−8^	<0.001
*GHRL* rs696217 (G)	Additive	2.01 (1.01–4.22); 0.046	2.11 (1.04–4.55); 0.037	0.084
	Dominant	0.71 (0.27–1.80); 0.469	0.79 (0.30–2.06); 0.630	0.69
	Recessive ^d^	40.8 (5.3–5217); 9.4 × 10^−6^	39.1 (5.0–4996); 1.8 × 10^−5^	<0.001

^c^ Adjusted for age, BMI and parity. ^d^ Homozygous class absent in controls (complete separation); Firth estimate is a penalised, descriptive value with a very wide interval. q: Benjamini–Hochberg across the nine adjusted model tests.

**Table 4:** Joint Multivariable Firth Logistic Regression Model.

Term	OR (95% CI)	*p*
*CYP19A1* rs2414096 (per A allele)	0.94 (0.52–1.66)	0.819
*GHRL* rs34911341 (per T allele)	2.75 (1.52–5.30)	0.0006
*GHRL* rs696217 (per G allele)	1.90 (0.82–4.57)	0.133
Age (per year)	1.04 (0.98–1.10)	0.189
BMI (per kg/m^2^)	1.05 (0.96–1.15)	0.304
Parity (per birth)	1.37 (0.89–2.15)	0.149

Model includes all three genetic variants (additive coding) simultaneously with clinical covariates. Firth’s penalised likelihood estimation was applied.

**Table 5:** Summary Table of in silico Analyses.

Gene	Polymorphism	Consequence/Region	Splicing Impact (SpliceAI/Pangolin)	Pathogenicity (CADD/PolyPhen/SIFT)	Protein Stability (I-Mutant)	Conservation (PhyloP)	Epigenomic State (RegulomeDB)	Overall Functional Verdict
*CYP19A1*	rs2414096	Intronic/Non-coding	No impact (Δ ≤ 0.00)	CADD: 2.6	N/A	0.846	Quiescent/Repressed Polycomb RegulomeDB 1f (0.55)	Benign/Non-disruptive
*GHRL*	rs696217	Missense (p.Leu72Met)	No impact (Δ ≤ 0.02)	CADD: 23.2 PolyPhen: 0.999 SIFT: 0.10	Decreased (RI = 8)	5.81	Weak transcriptional state RegulomeDB 1f (0.55)	Potentially deleterious/protein-destabilizing
*GHRL*	rs34911341	Missense (p.Arg51Gln)	No impact (Δ ≤ 0.01)	CADD: 25 PolyPhen: 0.998 SIFT: 0.05	Decreased (RI = 5)	5.98	Weak transcriptional state RegulomeDB 4 (0.61)	Potentially deleterious/predicted protein-destabilizing

CADD ≥ 20, PhyloP > 2, PolyPhen-2 ≥ 0.95, and SIFT < 0.05 were considered indicative of deleterious/conserved status. RI: Reliability Index (0–10). N/A: not applicable.

**Table 6:** Summary of the principal findings, their biological interpretation and limitations.

Finding	Principal Result	Biological Interpretation	Limitations
*CYP19A1* rs2414096	No difference in genotype or allele distribution between cases and controls. In silico: low CADD score, weak conservation, no predicted splicing effect, quiescent/Polycomb-repressed chromatin.	Aromatase-driven local estrogen synthesis is central to leiomyoma biology, but this intronic variant appears to be a benign, non-disruptive polymorphism, consistent with a Brazilian study of *CYP19*/*CYP17A1* [17]. PCOS associations [18,19] may reflect disease-specific (systemic hyperandrogenism) pathophysiology.	Limited power (65 cases, 72 controls); sparse AA genotype. The null result is inconclusive, not evidence of no association.
*GHRL* rs34911341 (Arg51Gln)	T allele associated with leiomyoma: adjusted OR = 2.85 (*p* = 0.0003; FDR q = 0.001). Sole independent genetic predictor in the joint model (OR = 2.75, *p* = 0.0006); independent of age, BMI and parity.	Arg51 is the C-terminal residue of mature ghrelin; substitution predicted to be deleterious (CADD, PolyPhen-2) and destabilising (I-Mutant) and could alter peptide processing. Compatible with the raised active-to-total ghrelin ratio reported in leiomyoma [20,21] and local ghrelin/GHS-R signalling [22].	TT genotype absent in controls (sparse cells, very wide CIs, risk of sparse-data bias); deviation from HWE and control allele frequencies above reference values (possible genotype misclassification); no Sanger validation; ghrelin not measured.
*GHRL* rs696217 (Leu72Met)	Nominal additive association (OR = 2.11, *p* = 0.037) not retained after FDR correction (q = 0.084) or in the joint model (OR = 1.90, *p* = 0.133). Genotype distribution differed; allele frequency did not.	Located in preproghrelin between ghrelin and obestatin; any effect likely concerns precursor stability or cleavage rather than mature ghrelin. Metabolic associations are inconsistent across populations [23,24,25].	Weak, non-robust signal; GG genotype absent in controls; same HWE and genotyping concerns; stability prediction refers to the precursor only.
Pathway level	Variant-specific findings only: *GHRL* rs34911341 associated, *CYP19A1* rs2414096 not.	No conclusion can be drawn on the relative importance of metabolic–endocrine versus steroidogenic pathways, or on interactions between them.	Only three candidate variants; no ghrelin or estradiol measurements; findings are exploratory and hypothesis-generating.
Population context	No previous study of these variants in leiomyoma in Turkish or neighbouring populations.	Other Turkish data show locus-specific effects (*GPER1*, *PON1*, *ACE*) [26,27,28]; *GHRL* 51Gln and 72Met frequencies vary markedly across regions [29,30,31,32].	Control frequencies exceed regional estimates; replication in independent Turkish cohorts with orthogonal genotyping is needed.

Abbreviations: CI, confidence interval; FDR, false discovery rate; HWE, Hardy–Weinberg equilibrium; OR, odds ratio; PCOS, polycystic ovary syndrome. Bracketed numbers refer to the reference list. Interpretations are hypothesis-generating; in silico predictions were not experimentally validated.

**Table 7:** Previous genetic and clinical studies relevant to the variants investigated.

Gene/Variant	Population and Condition	Main Finding	Ref.
A. Leiomyoma: steroidogenic and other candidate genes			
*CYP19*, *CYP17A1*	Brazilian women; leiomyoma	No difference in genotype or allele frequencies; steroidogenic polymorphisms unlikely to be major contributors to susceptibility	[17]
*GPER1* (GPR30) rs3808350, rs3808351	Turkish women; leiomyoma	rs3808351 G allele and GG genotypes of both variants associated with increased risk	[26]
*PON1* Q192R	Turkish women; leiomyoma	Heterozygous genotype associated with lower risk	[27]
*ACE* I/D	Turkish women; leiomyoma	No association	[28]
Ghrelin (active, total)	Women with leiomyoma vs. controls	Elevated active ghrelin with normal total ghrelin and obestatin; elevated active-to-total ratio	[20,21]
B. *CYP19A1* rs2414096 in other hormone-related conditions			
*CYP19A1* rs2414096	Chinese and Kashmiri women; PCOS	Associations with genotype distribution and hyperandrogenism markers	[18,19]
C. *GHRL* rs34911341 (Arg51Gln) and rs696217 (Leu72Met) in other phenotypes			
Arg51Gln	Obese individuals; middle-aged random sample	Originally identified in obesity; linked to lower plasma ghrelin and metabolic traits	[33,34]
Arg51Gln	Egyptian adults; type 2 diabetes	51Gln allele frequency 0.14 in controls vs. 0.27 in patients	[30]
Arg51Gln, Leu72Met	German children and students	51Gln ≈ 0.02; Met72 ≈ 0.09	[31]
Arg51Gln, Leu72Met	Han Chinese; metabolic syndrome	No 51Gln carriers; Met72 0.12–0.17; Leu72Met associated with metabolic syndrome	[32]
Leu72Met	Turkish–Cypriot; obesity	Associated with obesity	[29]
Leu72Met	Various populations; metabolic and cardiovascular traits	Met72 protective against NAFLD [35] but a risk factor for hypertension in type 2 diabetes [36]; meta-analysis conflicting (protective in Caucasians, increased susceptibility in Asians) [37]	[23,24,25]
Arg51Gln, Leu72Met	Chinese women; PCOS	No association	[38]
*GHRL* rs171407	European women (EPIC); breast cancer	Weak association with risk	[39]

Abbreviations: EPIC, European Prospective Investigation into Cancer and Nutrition; NAFLD, non-alcoholic fatty liver disease; PCOS, polycystic ovary syndrome. Allele frequencies refer to the minor allele reported in the cited study.

**Table 8:** PCR and RFLP conditions used for the polymorphisms of *CYP19A1* and *GHRL* genes.

Gene	Polymorphism	Primers	Annealing Temperature (°C)	Restriction Endonuclease	PCR Digestion Products (bp)
*GHRL*	rs34911341	P1 (F)/P2 (R)	65	SacI	C allele: 618 bp T allele: 455, 163 bp
*GHRL*	rs696217	P3 (F)/P4 (R)	53	BseNI (BsrI)	G allele: 618 bp T allele: 517, 101 bp
*CYP19A1*	rs2414096	P5 (F)/P6 (R)	60	Hsp92II	G allele: 189 bp A allele: 161, 28 bp

P1: F 5′CACAGCAACAAAGCTGCACC3′; P2: R 5′AAGTCCAGCCAGAGCATGCC3′; P3: F 5′GCTGGGCTCCTACCTGAGC3′; P4: R 5′GGACCCTGTTCACTGCCAC3′; P5: F 5′TCTGGAAACTTTTGGTTTGAGTG3′; P6: R 5′GATTTAGCTTAAGAGCCTTTTCTTCA3′. (F: forward primer, R: reverse primer).

## Data Availability

All data are available from the corresponding author upon reasonable request.
